# A Nudge-Based Campaign for Dementia Prevention Outcomes: A Pragmatic Cluster-Randomized Trial

**DOI:** 10.1093/geroni/igaf122.4279

**Published:** 2025-12-31

**Authors:** Jose Aravena, Hugo Castro, Ronald Poblete, Maria Aravena, Marilu Budinich, Patricio Fuentes, Cecilia Albala, Becca Levy

**Affiliations:** Yale University, New Haven, Connecticut, United States; CULTIVAMENTE, Santiago, Region Metropolitana, Chile; CULTIVAMENTE, Santiago, Region Metropolitana, Chile; CULTIVAMENTE, Santiago, Region Metropolitana, Chile; CULTIVAMENTE, Santiago, Region Metropolitana, Chile; Hospital Clinico Universidad de Chile, Santiago, Region Metropolitana, Chile; INTA, University of Chile, Santiago, Region Metropolitana, Chile; Yale School of Public Health, New Haven, Connecticut, United States

## Abstract

Although dementia prevention is a global priority, few interventions have been successfully translated into public health and community settings. To address this gap, we evaluated the effectiveness of a nudge-based Alzheimer’s disease (AD) prevention campaign on dementia prevention outcomes in older adults and healthcare providers. A pragmatic cluster-randomized trial was conducted in seven senior centers in Chile with adults aged ≥60 years who had cognitive impairment but no dementia. Centers were randomized to intervention (n = 3) or control (n = 4). All participants received usual care, including twice-weekly group activities (physical, cognitive, and social) and healthcare provider dementia training. Intervention centers additionally implemented CULTIVAMENTE, a nudge-based awareness campaign delivered through posters, brochures, and web content. We hypothesized that the intervention would improve cognitive healthy behaviors, cognitive performance (memory, executive function, and prevalence of mild-to-moderate cognitive impairment), and provider practices (AD prevention discussions and referrals). Intention-to-treat analyses using linear mixed models and generalized estimating equations confirmed these predictions. After six months, intervention participants (n = 101) demonstrated a 95.4% greater increase in cognitive healthy behaviors than controls (n = 110), significantly higher memory and executive function scores, and a lower prevalence of mild-to-moderate cognitive impairment. They were also more likely to report greater knowledge of AD prevention, to have engaged in discussions with providers about dementia risk reduction, and to have received referrals for managing risk factors. Embedding nudge-based messaging into senior centers improved dementia prevention behaviors, cognitive outcomes, and provider practices. These findings highlight the promise of low-cost, scalable strategies for advancing dementia prevention in real-world settings.

